# Postoperative Urinary Retention Following Thoracolumbosacral Spinal Fusion: Prevalence, Risk Factors, and Outcomes

**DOI:** 10.7759/cureus.19724

**Published:** 2021-11-18

**Authors:** Cheryl Marise Peilin Tan, Arun-Kumar Kaliya-Perumal, Glen Wen Kiat Ho, Jacob Yoong-Leong Oh

**Affiliations:** 1 Orthopaedic Surgery, Tan Tock Seng Hospital, Singapore, SGP; 2 Medicine, Lee Kong Chian School of Medicine, Singapore, SGP; 3 Orthopaedic Surgery, Yong Loo Lin School of Medicine, Singapore, SGP

**Keywords:** risk factors, trial off catheter, indwelling catheter, urinary retention, lumbar spine, spinal fusion, spine surgery

## Abstract

Objective

Postoperative urinary retention (POUR) is an often-underestimated common complication following spine surgery, and it is essential to avoid its untoward long-term consequences. Besides, a dilemma exists regarding the appropriate timing for the postoperative removal of indwelling catheter (IDC). Hence, we aim to describe the prevalence, risk factors, and outcomes of POUR and also come up with recommendations for the removal of IDC.

Methods

Electronic records of patients who underwent elective thoracolumbosacral spinal fusion surgery from January 2017 to December 2019 were retrospectively reviewed. Excluded were those who underwent fusion for indications such as trauma, cauda equina syndrome, infection, and malignancy. Both surgery-related and patient-related risk factors were tabulated, and their association with the likely development of POUR was assessed by univariate and multivariate analysis.

Results

One hundred sixty-eight patients (median age=64.1 years; 58.9% female) were included, with the incidence of POUR being 7.8%. Our findings suggest surgery-related factors, both intra- and postoperative, including operating time (p=0.008), anesthetic time (p=0.005), number of fusion levels (p<0.001), mobilization status prior to trial off catheter (TOC; p=0.021), and TOC timing (p=0.029) may have an association with POUR. In addition, patient-related factors, including the use of beta-blockers (p=0.020) and pre-operative mobility status (p<0.001), may also be associated with the likely development of POUR.

Conclusion

POUR seems to be a frequent complication following thoracolumbosacral spinal fusion surgery, which was found to have an association with some surgery-related and patient-related factors. While most of these factors are non-modifiable, certain modifiable risk factors provide the surgeon an opportunity to prevent POUR. Considering these factors, we recommend appropriate and timely mobilization of the patient prior to removal of IDC, which is to be performed preferably in the daytime.

## Introduction

Postoperative urinary retention (POUR) can be defined as the inability to void urine during the postoperative period despite a painful and distended bladder that is filled to capacity [1]. It is a relatively common complication following surgical procedures and causes discomfort [2], problems such as urinary tract infection [3], detrusor overdistention and damage [4], and ultimately, increased postoperative length of hospital stay [5]. The reported incidence of POUR following spinal surgery ranges between 5.6% and 39.4% [5-14]. This wide variation in the reported incidence may be due to the difference in the definition of POUR but is said to be influenced by factors such as pre-existing illness, mobility prior to surgery, chronic medications, and certain surgical parameters such as anesthesia, the operated region, approach, duration, and type of surgery [1].

Indwelling urinary catheters (IDCs) are routinely used during spine surgeries and are typically inserted just before the start of the surgery [6]. While most studies only report the routine placement of IDC during surgery, there is no clear consensus on the timing of its removal [7,8,12]. However, in general, it is considered that early removal of IDC may lead to POUR, while removing it late may pose a higher risk for urinary tract infection [15]. In order to gain further understanding of POUR and to come up with recommendations for postoperative removal of IDC, we retrospectively evaluated the data of those who had undergone elective thoracolumbosacral spinal fusion surgery at our institute with particular attention to prevalence, risk factors, and outcomes.

## Materials and methods

Study population

Electronic records of patients who underwent elective thoracolumbosacral fusion surgery at our tertiary care center (Tan Tock Seng Hospital, Singapore) over a three-year period from January 2017 to December 2019 were retrospectively reviewed. Included were those patients who failed conservative management and hence, required elective surgery. Patients who underwent emergency spinal fusion surgeries secondary to trauma, cauda equina syndrome, infection, or spinal malignancies were excluded. The study was approved by the Domain Specific Review Board (DSRB), National Healthcare Group (NHG), Singapore, and an exemption was granted (2020/00013).

Data collection

Preoperative variables collected were divided into patient-related and surgery-related factors (Table 1). Patient-related factors included age, gender, BMI, history of benign prosthetic hyperplasia (BPH), chronic constipation, diabetes mellitus (DM), smoking, urinary tract infection (UTI; diagnosed on an outpatient basis anytime within 90 days preoperatively), acute kidney injury (AKI), previous urinary retention, depression and use of beta-blockers. Surgery-related factors included duration of surgery, duration of anesthesia, intraoperative blood loss, surgical approach, spinal region operated, and number of spinal levels fused. In addition, postoperative variables including use of patient-controlled analgesia (PCA), length of stay, evidence of acute kidney injury (AKI), and urinary tract infection (defined by growth in the urine culture sample) were also collected.

**Table 1:** Surgery-related and patient-related variables included in the study PCA: patient-controlled analgesia; BO: bowel output; TOC: trial off catheter; BMI: body mass index; BPH: benign prostatic hyperplasia

Surgery-related variables (intra- and postoperative)	Patient-related variables (preoperative)
Operating time	BMI
Anesthetic time	Age
Length of stay	Gender
Blood loss	Prior urological malignancy
Approach	Prior BPH
Spinal regions operated	Prior BPH medications
Number of spinal levels operated	History of constipation
Intraoperative patient position	Diabetes mellitus
PCA use postoperatively	Smoking history
Acute kidney injury postoperatively	Preoperative urinary tract infection
Urinary tract infection postoperatively	Chronic kidney disease
Postoperative day patient was mobile	Prior urinary retention
Postoperative day trial off catheter (TOC)	Beta-blocker use
BO prior to TOC	Preoperative mobility
Mobilising prior to TOC	
TOC timing	

As a routine, all patients undergoing spinal fusion surgery had IDC inserted prior to the commencement of surgery. The postoperative day in which the IDC was removed was recorded. In addition, the postoperative day when mobilization was started with the physiotherapist was also recorded. POUR was considered established when re-insertion of the foley’s catheter was required after a trial of removal, following bladder distention, discomfort, failure to void, or a clinically significant post-void bladder volume of greater than 300 ml as confirmed by ultrasound scan.

Statistical analysis

Statistical analyses were performed using SPSS software ver. 22.0 (IBM, Armonk, New York). A probability (p) value of less than 0.05 was considered statistically significant. Logistic regression was used to analyze the association between the development of POUR and risk factor variables, both patient-related and surgery-related. Univariate assessment, followed by multivariate regression analysis, were performed to identify independent risk factors.

## Results

**Figure 1:**
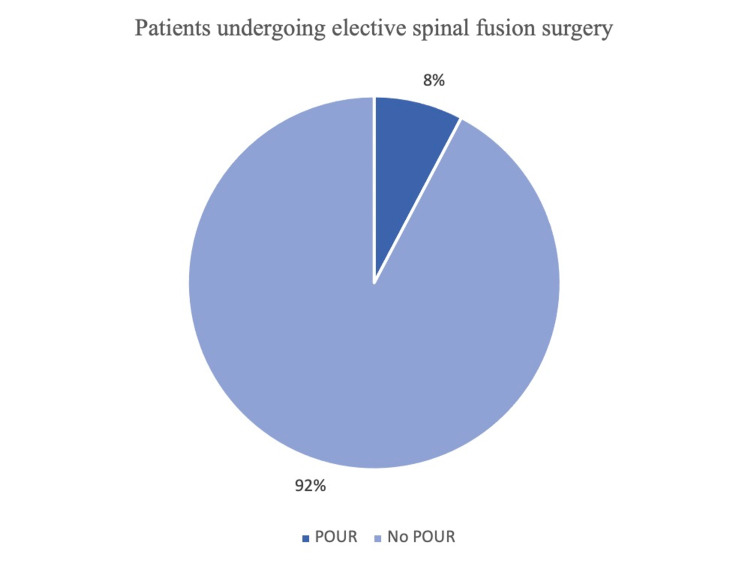
Percentage of patients with POUR post elective spinal fusion surgery

A total of 168 patients were included in this study. In this patient population, the incidence of POUR was found to be 7.8% (13/168) (Figure 1). The mean age at surgery was 64.1 years, and 58.9 % of patients were female. The mean body-mass index (BMI) was 26.8 kg/m2. Based on univariate analysis (Table 2), it was found that preoperative use of beta-blockers and mobility status had a positive association with POUR, with significance levels of 0.020 and < 0.001 respectively (odds ratio [OR] = 4.000 for beta-blockers). Besides, surgery-related factors such as operating time (OR = 0.999; 95% CI; p=0.008), anaesthetic time (OR = 0.997; 95% CI; p=0.005), number of spinal levels (p < 0.001), whether the patient was mobilizing prior to trial off catheter (TOC) (p = 0.021) and TOC timing (OR = 0.206; 95% CI; p = 0.029) were also found to have an association with POUR (Table 3).

**Table 2:** Demographics and univariate analysis showing association of patient factors with postoperative urinary retention (failing 1st trial off catheter) TOC: trial off catheter; BMI: body mass index; BPH: benign prostatic hyperplasia; DM: diabetes Mellitus; ADL: activities of daily living

	Total, n=168	Fail TOC		p-value	Odds ratio
		Yes (%)	No (%)		
BMI, kg/m^2^, mean	26.83	28.69	26.67	0.125	0.910
Age, years, mean	64.08	67.54	63.79	0.204	0.955
Gender					
Male	69	5 (7.2)	64 (92.8)	1.000	0.889
Female	99	8 (8.1)	91 (91.9)		
Prior urological malignancy					
Yes	1	0 (0)	1 (100)	1.000	1.006
No	167	13 (7.8)	154 (92.2)		
Prior BPH					
Yes	13	1 (7.7)	12 (92.3)	1.000	0.993
No	155	12 (7.7)	143 (92.3)		
Prior BPH medications					
Yes	13	1 (7.7)	12 (92.3)	1.000	0.993
No	155	12 (7.7)	143 (92.3)		
History of constipation					
Yes	1	1 (100)	0 (0)	0.077	0.923
No	167	12 (7.2)	155 (92.8)		
DM					
Yes	49	5 (10.2)	44 (89.8)	0.526	1.577
No	119	8 (6.7)	111 (93.3)		
Smoking					
Yes	37	2 (5.4)	35 (94.6)	0.736	0.623
No	131	11 (8.4)	120 (91.6)		
Preoperative urinary tract infection					
Yes	2	0 (0)	2 (100)	1.000	1.013
No	166	13 (7.8)	153 (92.2)		
Chronic kidney disease					
Yes	12	0 (0)	12 (100)	0.602	1.084
No	156	13 (8.3)	143 (91.7)		
Prior urinary retention					
Yes	9	1 (11.1)	8 (88.9)	0.522	1.531
No	159	12 (7.5)	147 (92.5)		
Beta-blocker use					
Yes	42	7 (16.7)	35 (83.3)	0.020	4.000
No	126	6 (4.8)	120 (95.2)		
Preoperative mobility					
ADL-independent	160	10 (6.3)	150 (93.8)	< 0.001	
ADL-assisted	5	1 (20.0)	4 (80.0)		
Wheelchair mobility	3	2 (66.7)	1 (33.3)		

**Table 3:** Univariate analysis showing association of surgical factors with postoperative urinary retention (failing 1st trial off catheter) TOC: trial off catheter; PCA: patient-controlled analgesia; BO: bowel output; *Excluded from analysis in view of minimal patient number

	Total, n=168	Fail TOC		p-value	Odds ratio
		Yes (%)	No (%)		
Operating time, mean, minutes	354.89	448.46	347.05	0.008	0.999
Anesthetic time, mean, minutes	450.55	556.00	441.70	0.005	0.997
Length of stay, mean, days	9.70	12.54	9.46	0.067	
Blood loss					
0-500ml	137	8 (5.8)	129 (94.2)	0.066	
>500 ml	31	5 (16.1)	26 (83.9)		
Approach					
Posterior	135	10 (7.4)	125 (92.6)	0.839	
Lateral & Posterior	31	3 (9.7)	28 (90.3)		
Anterior & Posterior	2	0 (0)	2 (100)		
Spinal regions operated					
Lumbar	113	6 (5.3)	107 (94.7)	0.266	
Thoracolumbar	1*	0 (0)	1 (100)		
Lumbosacral	50	6 (12.0)	44 (88.0)		
Thoracolumbosacral	4	1 (25.0)	3 (75.0)		
Number of spinal Levels					
1-2	73	2 (2.7)	71 (97.3)	< 0.001	
3-4	76	5 (6.6)	71 (93.4)		
≥5	19	6 (31.6)	13 (68.4)		
Intraoperative patient position					
Prone	136	10 (7.4)	126 (92.6)	0.814	
Lateral & prone	30	3 (10.0)	27 (90.0)		
Supine & prone	2	0 (0)*	2 (100)		
PCA use postoperatively					
Yes	128	9 (7.0)	119 (93.0)	0.509	0.681
No	40	4 (10.0)	36 (90.0)		
Acute kidney injury postoperatively					
Yes	4	0 (0)	4 (100)	1.000	1.026
No	164	13 (7.9)	151 (92.1)		
Urinary tract infection postoperatively					
Yes	6	2 (33.3)	4 (66.7)	0.069	6.864
No	162	11 (6.8)	151 (93.2)		
Postoperative day patient was mobile					
Early (1-2)	105	5 (4.8)	100 (95.2)	0.077	0.344
Late (3 onwards)	63	8 (12.7)	55 (87.3)		
Postoperative day trial off catheter (TOC)					
Early (0-1 days)	12	1 (8.3)	11 (91.7)	0.400	
Normal (2-7 days)	145	10 (6.9)	135 (93.1)		
Late (>7 days)	11	2 (18.2)	9 (81.8)		
BO prior to TOC					
Yes	86	7 (8.1)	79 (91.9)	1.000	1.122
No	82	6 (7.3)	76 (92.7)		
Mobilizing prior to TOC					
Walking	154	10 (6.5)	144 (93.5)	0.021	
Standing	5	2 (40.0)	3 (60.0)		
Lying in bed	9	1 (11.1)	8 (88.9)		
TOC timing					
Day	151	9 (6.0)	142 (94.0)	0.029	0.206
Night	17	4 (23.5)	13 (76.5)		

**Figure 2:**
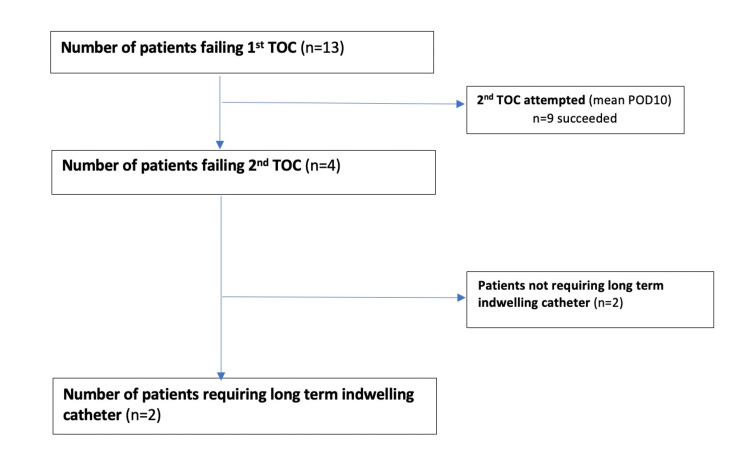
Breakdown of patients failing 1st and 2nd trial off catheter TOC: trial off catheter

Multivariate regression analysis was performed to analyze whether those patient-related and surgery-related risk factors that were found to be significant in our univariate analysis were independent risk factors for POUR. However, our results indicate that none of them are independent risk factors. Of the 13 patients who failed the first TOC and diagnosed to have POUR, only one was noted to have a urinary tract infection. On average, a second TOC was attempted on postoperative day 10. In one patient, a second TOC was not attempted, and he was placed immediately on a long-term catheter. Four out of 12 patients failed the second TOC, and two patients required long-term IDC while the remaining 2 patients eventually managed the TOC at a later date (Figure 2).

Overall, patient-related factors such as the use of beta-blockers and non-ADL independent preoperative ambulatory status increased the risk of POUR. In addition, a longer operating and anaesthetic time, more spinal levels operated on, poorer mobilization prior to TOC (standing or lying in bed), and the removal of an IDC at night showed a positive association with POUR.

## Discussion

POUR is an often-underestimated common complication following spine surgery. There is wide variability in how authors assess the incidence of POUR [5-14]; for instance, Golubovsky et al. [12] studied POUR only in patients undergoing posterior lumbar surgery, whereas Altschul et al. [7] included all patients undergoing cervical, thoracic or lumbar surgeries using different approaches. Moreover, different definitions of POUR were also used. For example, Atschul et al. [7] defined POUR as the inability to void urine for more than eight hours post TOC in spite of a painful and distended bladder, whereas Lee et al. [8] defined it as an inability to void or a having a residual post-void volume of more than or equal to 100 ml, two days post-operation. In our study, POUR was considered established when re-insertion of the Foley catheter was required after a trial of removal, following bladder distention, discomfort, failure to void, and a clinically significant post-void bladder volume of greater than 300 ml as confirmed by ultrasound scan. 

The incidence of POUR in our study was 7.8%, well within the range noted in literature [5-11,13,14]. Confounders were limited by examining only patients undergoing elective thoracolumbosacral spinal surgery, excluding emergency spinal fusion surgeries secondary to trauma, cauda equina, or spinal malignancies. We collected accurate data on the post-void residual urine (PVRU) and timing of TOC. Since there are no established guidelines regarding the appropriate timing for removal of IDC, we usually perform TOC depending upon the patient's mobilization status post-surgery, which, by itself, depends on the type and extent of surgery performed and patient's tolerance to physiotherapy as deemed by the surgeon. Furthermore, we could categorize patients as those who failed 1st TOC (category 1), failed 2nd TOC (category 2), and required long-term catheter (category 3). With a minimum postoperative follow-up of up to one year, we were able to study the prognosis in all these categories of patients. This helped to provide better accuracy in estimating and studying the outcomes of POUR following selective procedures at our institution.

POUR is associated with potential complications, adding up to healthcare costs and additional patient burden following spine surgery [16-18]. This study was thus conducted with the aim of understanding risk factors and developing recommendations for the postoperative removal of IDC. Based on prior literature [5-8, 12-14], we hypothesized that patient-related factors such as older age, male gender, higher BMI, BPH, preoperative UTI, use of beta-blockers, and mobility status were risk factors for developing POUR. We further hypothesized that patients undergoing lumbar spinal fusion surgery with a longer operating or anesthetic time were at a higher risk for developing POUR.

Our results did show that the preoperative use of beta-blockers and mobility status had an association with the development of POUR. In addition, surgery-related factors such as longer operating time and anaesthetic time also showed a positive association. These factors were in line with our initial hypothesis. In addition, multi-level surgeries had an association with POUR; however, this may not be independent and could be influenced by factors such as operating or anesthetic time. Similar to our findings, various studies have reported that patients undergoing prolonged surgeries have an increased risk of POUR. Atschul et al. [7] conducted a retrospective review of 397 patients and observed the mean operative time to be 167 minutes in those who did not develop POUR and 213 minutes in those who did develop POUR - a difference that was statistically significant. The same was observed in various studies [8,12,13,14]. 

Interestingly, our study shows a positive association between beta-blocker use and the development of POUR. This was in line with Boulis et al.'s [6] study, wherein they noted that preoperative beta-blockers contributed to a higher incidence of urinary retention. As the literature on beta-blocker use and POUR is limited, it is a potential area for further study, which may influence our management of POUR and the protocol for TOC in patients using beta-blockers. Preoperative mobility status and its association with POUR were not well-described previously. However, we found that the preoperative mobility status (classified into activities of daily living (ADL)-independent, ADL-assisted, and wheelchair mobile) has an association with POUR (p < 0.001). This may also explain why poor mobilization prior to TOC showed a positive association with POUR. 

Based on our findings, recommendations for the postoperative removal of IDC may be extrapolated: to ensure mobilization (walking) prior to TOC (p = 0.021) and performing TOC in the day instead of the night (p = 0.029). However, it should be noted that association need not necessarily represent causation, and further studies are required to assess the strength and consistency of the association that was identified here. In addition, being a retrospective study, there are certain limitations. With a smaller number of patients included, our study may be lean to draw potential conclusions; however, we believe it to be of clinical importance and can be considered as a pilot analysis to estimate risk factors. Further, based on our definition of POUR, we may have under or overestimated the incidence of POUR as compared to other studies.

## Conclusions

A retrospective study was conducted to describe the prevalence, risk factors, and outcomes of POUR following elective thoracolumbosacral spinal fusion surgeries. Based on our study, the incidence of POUR was found to be 7.8%. Our findings suggest surgery-related factors including operating time, anesthetic time, number of fusion levels, mobilization status prior to TOC, and TOC timing may be associated with the likely development of POUR. In addition, patient-related factors, including the use of beta-blockers and pre-operative mobility status, could also be associated with the occurrence of POUR. Among these, the modifiable risk factors give an opportunity to prevent POUR before it ensues. Notably, appropriate and timely mobilization of the patient prior to removal of IDC and removal of IDC in the daytime may prevent POUR.
